# Factors Influencing Rapid Antiretroviral Therapy Initiation at Four eThekwini Clinics, KwaZulu-Natal, South Africa

**DOI:** 10.1007/s10461-021-03530-3

**Published:** 2021-11-15

**Authors:** Sabina M. Govere, Chester Kalinda, Moses J. Chimbari

**Affiliations:** 1 School of Nursing and Public Health, Discipline of Public Health Medicine, University of KwaZulu-Natal, Mazisi Kunene Road, Glenwood, Durban, 4041 South Africa; 2 School of Science, University of Namibia, Katima Mulilo Campus, Box 1096, Ngweze, Katima Mulilo, Namibia

**Keywords:** Universal test and treat, Rapid ART initiation, Same-day ART initiation, Antiretroviral therapy

## Abstract

Timely uptake of Antiretroviral therapy considerably improves the health of people living with the Human Immunodeficiency virus. We conducted a cross-sectional study of newly HIV diagnosed individuals in four clinics in eThekwini municipality, KwaZulu-Natal. Data was collected between June 2020 and December 2020. Participants completed an interviewer-administered questionnaire after HIV testing, on the day of HIV diagnosis. We evaluated factors influencing uptake of same-day ART initiation in eThekwini clinics, KwaZulu Natal, South Africa. Demographic information, health status, sexual behaviour, knowledge of universal test and treat (UTT), ART initiation uptake, and disclosure data was collected. Among the 403 participants, same-day initiation (SDI) was 69.2% (n = 279). We observed the number of sexual partners (aOR 0.35; 95% CI 0.15–0.81), HIV status of the partner (aOR 5.03; 95% CI 2.74–9.26) and knowledge of UTT (aOR 1.97; 95% CI 1.34–2.90) were identified as major factors influencing uptake of same-day ART initiation. More strategies are needed to achieve the SDI uptake within the framework of UTT.

**Supplementary Information:**

The online version contains supplementary material available at 10.1007/s10461-021-03530-3.

## Background

The World Health Organization (WHO) defines rapid initiation of antiretroviral therapy (ART) as the commencement of highly active antiretroviral therapy (HAART) on the same day of HIV diagnosis [1]. Substantial progress has been observed in recent years in the roll-out of antiretroviral therapy (ART) to populations in sub-Saharan Africa [2]. WHO also recommends ART initiation on the same day as HIV diagnosis, after ensuring the person’s willingness and readiness to start ART unless there are clinical reasons to delay treatment [1]. Global effort on decreasing continuous HIV transmission emphasizes the need for routine HIV testing and timely uptake of ART [3]. Advanced, cost‐effective, and scaling up strategies are needed to meet the Joint United Nations Program on AIDS/HIV (UNAIDS) ambitious 95‐95‐95 goals: 95% of people living with HIV (PLHIV) know their serostatus, 95% of those are on sustained (ART) and 95% of those are virally suppressed [4]. Whilst focussing on multi-faceted prevention measures in areas with hyper endemic HIV infection, intensified provision of ART is seen as an important component with a direct impact on population HIV transmission [5, 6].

South Africa carries about 17% of the world's HIV-positive population [2, 7] making it the heaviest carrier of the global HIV burden. Within South Africa, KwaZulu-Natal province is the worst affected having about 1.6 million HIV-positive individuals and over 50% prevalence between the ages 15 to 25 years [4]. Despite the well-known need for protection from HIV infections and other reproductive health risks, their age and their social and economic status limit access to information and services in many settings. Adolescence and young adulthood is typically a period of experimentation, new sexual experiences, and vulnerability. Some adolescents and young adults may experiment with injecting drugs, sexuality, and sexual orientation (men may begin to have unprotected sex with other men), and some are exploited sexually [8]. To shrink continuous HIV transmission, South Africa adopted the universal test and treat (UTT) policy for eligible individuals in September 2016 [8]. The UTT policy aims to reduce HIV infection through expanding prevention and treatment preferences. UTT was evaluated in four randomized population-based trials (BCPP/Ya Tsie, HPTN 071/PopART, SEARCH, ANRS 12249/TasP) conducted in sub-Saharan Africa (SSA) resulting in formulation of World Health Organization guidelines and the UNAIDS 90-90-90 campaign.” [5]. By June 2019, an estimated 91% of people living with HIV (PLHIV) knew their HIV infection status [4] and of these, 68% were on ART and 83% had viral suppression To improve ART initiation and retention especially among men who have been observed to have poorer treatment uptake, retention and viral suppression compared to women living with HIV [3, 10] there is need to have targeted interventions. HIV prevalence in eThekwini District accounts for 17% of KwaZulu-Natal’s prevalence. The district reported 82% of people testing positive for HIV, 72% of those diagnosed with HIV were initiated on ART and 68% of those initiated on treatment were found to be virally suppressed [18]. These results suggest a gap in reaching the 90-90-90 targets.

It is crucial to understand the complexity of factors influencing a person's decision to get on treatment following diagnosis. One of the most persistent challenges facing ART programs in Africa is the late presentation for testing as well as high rates of attrition between HIV testing and ART initiation [11]. Loss to care before starting ART has constantly been high among diagnosed individuals that are eligible for treatment [12]. The recommendation by WHO to offer ART to all who test positive regardless of CD4 count and clinical staging has little benefit if people who test positive fail to initiate treatment immediately [13]. Loss of ART-eligible clients before treatment initiation experienced in many sub-Saharan African countries including South Africa [14] can be addressed through SDI.

The reasons for an individual’s rapid or deferred ART uptake may be complex and unintentional. Previously in the HIV cascade of care, there was a gap between having an HIV test and initiating ART. Individuals had to provide CD4 count blood samples and returning for results [24]. Under the new guidelines, CD4 counts will no longer be required to establish ART eligibility to reduce the loss HIV diagnosed individuals at this stage. Losses at this stage were likely related to both health system barriers, such as requirements for multiple clinic visits and delays in receiving laboratory test results, and to patient factors; such as time constraints or reluctance to commit to lifelong treatment [24]. Although several observational studies on SDI in Sub-Saharan African settings have been conducted, the focus was mainly on pregnant and breastfeeding women hence the results may therefore not be generalizable to the broader adult population [15]. Other studies have been restricted to hospitals settings [16] or have described SDI in the context of specialized interventions such as peer-delivered linkage case management or have been implemented at a single facility [17]. There is therefore paucity of data on the uptake of SDI by the adult population in high HIV prevalence settings. It is against this background that we conducted this study which aimed to identify factors affecting uptake of same-day ART initiation in eThekwini clinics.

## Methodology

### Study Design and Setting

This prospective cross-sectional study was conducted at 4 clinics in eThekwini municipality KwaZulu-Natal (KZN), South Africa between June 2020 to December 2020. The study sites were *Ithembalabantu*, Pinetown, D, and *Qadi* clinic. KZN has 1.9 million people living with HIV of which only 1.1 million are on ART [18]. Of the estimated 650,000 people living with HIV in eThekwini, there are approximately 383,869 people in the ARV program [4]. The eThekwini district is densely populated (3,702,231) comprising of urban, semi-urban, and rural areas [11]. We selected study clinics from the three settings; (i) 2 facilities (*Ithembalabantu* and D clinic) in a densely populated Umlazi township also known to have high HV prevalence, (ii) Pinetown clinic in Pinetown, a semi suburb town (these are places that offer a balance between township and suburb tranquillity) surrounded by townships and informal settlements who all seek services at the facility and (iii) *Qadi* clinic in rural *Umzinyathi* district municipality north of eThekwini municipality. UMzinyathi district has high levels of poverty, unemployment, and HIV/AIDS [18].

### Study Population and Data Collection

Four hundred and sixty-one individuals seeking voluntary HIV testing and counseling were recruited for the study. We included newly HIV-diagnosed adults (18 years or older) who presented for voluntary HIV counselling and testing between June to December 2020. Eligible participants were enrolled in the study after self-reporting that they have never tested positive for HIV before. Selection bias was possible if participants decide to report not having an HIV-positive diagnosis before yet they are aware they are HIV positive and have actually taken ART before. After explaining the study to the participants, obtaining written consent, and excluding individuals previously on antiretroviral therapy (ART), 403 adults were included in the study.

### Data Collection

The participants were first tested for HIV by clinic HIV Counselling and Testing Counsellors who were non-research staff members. Individuals who tested HIV positive were referred to a research assistant for enrolment irrespective of accepting same-day ART initiation or different ART. Participants completed an interviewer-administered baseline questionnaire after HIV testing and either received ART initiation or not, on the day of HIV diagnosis. Initiation of ART was also confirmed using prescription cards in the medical charts. A qquestionnaire was prepared in the local language *IsiZulu* and was administered to participants at the clinic through face-to-face interviews by a team of two community research assistants (CRAs) supervised by the investigator. The CRAs were native *IsiZulu* speakers and had post-high school qualifications and were trained on the study including the data collection process. The questionnaire captured participant’s demographic information (age gender, marital status, employment status, education level), health status, and sexual behaviour history (number of current sexual partners, status of sexual partner, perceived risk of HIV infection) knowledge of UTT, ART initiation uptake as well as disclosure data. The questionnaire was pre-tested on a sample of 6 participants from the exact target group at Ithembalabantu clinic.

Data were collected electronically using the Kobo Collect application (Cambridge, MA, USA) on Android mobile devices. Non-research trained HIV testing counselors conducted HIV tests according to the South African guidelines [19]. We enrolled approximately 100 participants in each of the four clinics including individuals who rapidly initiated ART and those that delayed. The same-day initiation (SDI) of ART was defined as acceptance of ART on the day of HIV diagnosis and given prescribed medication (ART) on the same day. We used prescription cards in the medical charts to confirm and check ART initiation. At the time of enrolment, there were changes on ART regimen guidelines in South Africa. Dolutegravir (DTG), an integrase inhibitor drug was introduced as part of fixed-dose combinations of Tenofovir, Lamivudine, and Dolutegravir (TLD) from Tenofovir, Emtricitabine, and Efavirenz (TEE). With a high genetic barrier to resistance, DTG has the potential to curb the spread of antiretroviral resistance, as it is highly effective, well-tolerated, and affordable in resource-limited settings. All healthcare facilities offering ART in South Africa are recommended to initiate individuals on DTG unless pregnant or women of child-bearing age are not willing to take DTG due to fear of tubal defects.

### Ethical Considerations

This study was approved by the University of KwaZulu-Natal Biomedical Research Ethics Committee (BREC/00000819/2019).

### Patient and Public Involvement

The participants and general public were not involved in the development of the research question, outcome measures, design, recruitment, and conduct of this study.

### Data Analysis

Data cleaning and analysis were done using Stata SE version 17 [20]. Summary statistics including frequencies were used to describe the characteristics of the study subjects. The variable of interest was ART initiation time. South Africa has adopted a UTT and SDI of antiretroviral therapy [7]. Thus, for our study, we have SDI and not same-day initiation (NSDI). This was converted into a dichotomous variable. In our analysis, we determined the relationship between socio-demographic characteristics and the response variable using the Pearson chi-square test and a Fisher's Exact test when frequencies were small. Variables that were significant in the Pearson chi-square test as well as those that had the p-value of < 0.20 were included in the univariate analysis. We fit a multivariate logistic regression model to examine the relationship between several explanatory variables and ART initiation time. The estimated odds ratio with their 95% confidence interval was used to determine the strength of association and significant variables were identified.

To test the goodness of fit for the final model, the Hosmer Lemeshow test was applied. The analysis also explored clustering of the health facilities. However, the ICC (intra-class correlation coefficient) estimated from this data was 0.046 indicating that there would be no need for a multilevel model that would take into consideration variations among the health facilities [21].

## Results

Of 461 individuals invited to participate in the study, 403 agreed to take part (response rate = 87.3%). Only participants who provided complete information relating to variables of interest were included in the final analytical sample. Participant characteristics of the sample and unadjusted associations between the response variable and the socio-demographic characteristics of participants are presented in Table 1. The mean (SD) age of respondents was 32.7 (± 9.1). The majority (42.7%, n = 172) of the respondents were aged between 29 and 39 years and 63.1% (n = 254) were females. The overall prevalence of SDI was 69.2% (n = 279) (Table 1). The study also showed that 47.3% (n = 140) of participants on SDI were single. The median number of children that the participants had was 2 (IQR = 1). Our results suggest that age, marital status, education, education level, employment status, number of biological children, knowledge of UTT, number of current sexual partners, the HIV status of the sexual partners and risk of infection prior to testing were significantly associated (*p* < *0.05*) with SDI of antiretroviral therapy. Gender was the only variable which was not significantly associated with SDI (Table 1).

**Table 1:** Descriptive statistics for study variable

Variables	Same-day initiation (SDI)		Not same-day initiation (NSDI)		p-value
	Freq (n)	%	Freq (n)	%	
*Age*					
18–28	73	49.3	75	50.7	0.001*
29–39	145	84.3	27	15.7	
40–50	43	70.5	18	29.5	
51–62	18	81.8	4	18.2	
*Gender*					
Female	177	69.7	77	30.3	0.796
Male	102	68.5	47	31.5	
*Marital status*					
Cohabiting	56	76.7	17	23.3	0.001*
Divorced	5	45.5	6	54.5	
Married	66	83.5	13	16.5	
Single	132	60.6	86	39.4	
Widowed	20	90.9	2	9.1	
*Education*					
Primary	67	68.4	31	31.6	**0.002**
High school	119	62.3	72	37.7	
Tertiary	93	81.4	21	18.4	
*Employment status*					
Employed	104	83.2	21	16.8	**0.001**
Self employed	19	61.3	12	38.7	
student	24	51.1	23	48.9	
Unemployed	132	66	68	34.0	
*Biological children*					
No	54	61.4	34	38.6	**0.071**
Yes	225	72.4	90	28.6	
*Knowledge of UTT*					
No	137	56.6	105	43.4	0.001*
Slightly	46	79.3	12	20.7	
Yes	96	93.2	7	6.8	
*Number of current sexual partners*					
One	75	90.4	8	9.6	**0.001**
More than 2	204	63.8	116	36.2	
*HIV status of sexual partner*					
Negative	4	28.6	10	71.4	0.001*
Unknown	130	55.6	104	44.4	
Positive	145	93.6	10	6.4	
*Infection risk prior to testing*					
Definitely not going to acquire HIV	15	50.0	15	50.0	**0.001**
Probably not going to acquire HIV	73	49.7	74	50.3	
Probably will become Infected	40	61.5	25	38.5	
Definitely will become positive	150	93.2	11	6.83	

*Fisher’s test conducted die to small frequencies in some cells

### Initiation on First-Line Antiretroviral Regimen

Participants who accepted same day initiation (SDI), 33.0% (n = 94) opted not to be initiated on the new first-line drug Tenofovir 300 mg, Lamivudine 300 mg and Dolutegravir 50 mg (TLD) preferring to be initiated on Tenofovir 300 mg Emtricitabine 200mh and Efavirenz 600 mg (TEE). In addition, the study showed that among those who were not initiated on TLD, 95.7% (n = 90) were females while 4.3% (n = 4) were males.

### Univariate and Multivariate Analysis of Same-Day Initiation

In a univariate analysis, age of respondent (OR: 0.49, 95% CI 0.36–0.67), marital status (OR: 1.27, 95% CI 1.04–1.56), employment status (OR: 1.21, 95% CI 1.06–1.39), education (OR: 1.39, 95% CI 1.03–1.87), number of current sexual partners (OR: 0.18, 95% CI 0.08–0.40), HIV status of the sexual partner (OR: 8.75, 95% CI 4.91–15.59), infection risk before testing (OR: 0.514, 95% CI 0.41–0.64), knowledge of UTT (OR: 3.17, 95% CI 2.21–4.55), gender (OR: 0.94, 95% CI 0.61–1.46), and having biological children (OR: 1.57; 95% CI 0.96–2.57) were significantly associated with SDI (Table 2).

**Table 2:** Univariate and multivariate analysis for factor influencing same-day initiation

Determinant	OR (unadjusted)	95% CI	aOR (adjusted)	95% CI
*Age*				
18–28	Reference			
29–39	**5.52**	**3.27–9.30**		
40–50	**2.45**	**1.29–4.64**		
51–62	**4.62**	**1.49–14.31**		
*Marital status*				
Cohabiting	Reference			
Divorced	**0.25**	**0.068–0.93**		
Married	1.54	0.69–3.45		
Single	**0.46**	**0.25–0.85**		
Widowed	3.03	0.64–14.32		
*Education*				
Primary	Reference			
High school	0.76	0.45–1.28		
Tertiary	2.05	1.08–3.87		
*Employment status*				
Employed	Reference			
Self Employed	**0.319**	**0.14–0.76**		
Student	**0.21**	**0.10–0.44**		
Unemployed	**0.39**	**0.22–0.68**		
*Knowledge of UTT*				
No	Reference			
Slightly	**2.94**	**1.48–5.82**	**1.21**	**0.55–2.81**
Yes	**10**	**4.68–23.58**	**4.39**	**1.82–10.65**
*Number current of sexual partners*				
One	Reference			
More than 2	**0.19**	**0.09–0.40**	**0.35**	**0.15–0.81**
*HIV status of sexual partner*				
Negative	Reference			
Unknown	3.12	0.95–10.25	**2.18**	**1.02–6.92**
*Positive*	**6**	**4.63–13.35**	**4.83**	**5.43–8.77**
*HIV Infection risk prior to testing*				
Definitely not going to become HIV positive	Reference			
Probably not going to become HIV positive	1.01	0.46–2.22		
Probably will become HIV positive	1.6	0.67–3.83		
*Definitely will become HIV positive*	**13.6**	**5.31–34**		

In a multivariate analysis, the number of sexual partners (aOR 0.35; 95% CI 0.15–0.81), the HIV status of the partner (aOR 5.03; 95% CI 2.74–9.26) and knowledge of UTT (aOR 1.97; 95% CI 1.34–2.90) were the factors that influenced SDI. We observed that the likelihood of SDI among participants with more than one sexual partner were 0.35 (95% CI 0.15–0.81) fold lower than those who had one sexual partner. On the other hand, the likelihood of SDI for those who didn’t know the HIV status of their partners and those whose partners were HIV positives were 2.18 (95% CI 1.02–6.92) and 4.83 (95% CI 5.43–8.77) fold higher, respectively compared to those whose partners were HIV-negative. We also observed that those who had knowledge on UTT were 4.39 (95% CI 1.82–10.65) times more likely to initiate SDI compared to those who had no knowledge. No differences in initiation of SDI were observed between those who were slightly knowledgeable and those who had no knowledge about UTT (Table 2).

## Discussion

This study found that the magnitude of same-day ART initiation demonstrated some progress in the uptake of test and treat programs in eThekwini clinics. The findings are comparable with those obtained from a study conducted by Koenig et al. where an SDI prevalence of 57% [22] was reported. The results obtained from the current study suggest the need for increased community sensitization on the benefits of SDI for clients who are HIV positive.

In sub-Saharan Africa, only about 26–37% of persons diagnosed with HIV have been observed to enrol in care and immediately initiate ART when provided with standard referral services [17]. Elsewhere, studies have shown that Italy reported a 78% progress in reaching the “second 95” in SDI [23] while Uganda which has a high HIV prevalence reported 71% SDI [24]. In contrast, our results from eThekwini, as well as those reported from a study conducted in Johannesburg show that 65.9% of individuals initiated ART under UTT [7]. Altogether, our results from South Africa suggest the need for enhanced efforts to reach the “second 95” of the UNAIDS 95‐95‐95 targets [4, 25] by 2030 on timely initiation of ART to realize a meaningful change in ART initiation coverage.

Our results suggest that gender does not influence the uptake of SDI. This outcome is similar to the findings from a study conducted in Zimbabwe [16]. However, our results on the influence of gender on SDI contrast the observations made by Lilian et al. These authors suggested that men tend to take longer to accept a positive diagnosis. The prevailing hegemonic masculinity in the study area which suggests that males must be dominant over females and must exhibit physical and emotional toughness, strength and endurance [17, 35] may explain the men’s poorer outcomes to HIV care and treatment when compared to women. Their results suggest that denial was high in males compared to females. Other studies observed that men were less likely to initiate treatment when diagnosed with HIV [19, 26]. In eThekwini where the current study was conducted, an earlier study concluded that men presented late for HIV testing [27]. The late presentation of men is likely due to work demands that keep them from seeking treatment earlier in the course of the disease.

We observed that behavioural factors such as self‐reported sexual behaviour relating to the number of current sexual partners and HIV status of the sexual partners influenced SDI. For instance, we observed that participants with more than one sexual partner were less likely to start ART immediately. Fear of disclosing and losing the partners might be contributing factors to delay in SDI. Furthermore, individuals with multiple sexual partners were less likely to rapidly initiate ART increasing the risk of infecting others as well as re-infecting their partners [31, 32]. It is crucial for HIV-positive individuals engaging in multiple sexual partner relationships to immediately initiate ART to prevent a chain of transmission and reinfection. We further observed that participants who didn’t know the HIV status of their partners and those whose partners were positive were more likely to initiate SDI. Immediate initiations of ART for these groups especially those with HIV positive partners is essential among people with HIV-positive partners may be a way of early reduction of the viral load [30]. It is crucial for HIV positive individuals engaging in multiple sexual partner relationships to immediately initiate ART to prevent a chain of transmission and reinfection.

Our results from the multivariate analysis indicates that self‐acknowledged risk perception of HIV infection did not influence SDI uptake. The way PLHIV process a positive result can influence their engagement with HIV treatment and care. Available evidence suggests that acceptance of HIV status is exacerbated by associating oneself with HIV through judgments about sexual behaviours, which shape a sense of personal risk of infection [30]. One possible explanation from our findings is that the SDI individuals were more willing to acknowledge living with HIV and hence initiated SDI more readily. In a study conducted in Swaziland, people who reported few sexual partners felt they were not at risk of HIV infection and struggled to accept a positive result thus delaying ART initiation [34]. Perception of HIV risk continues to have associations with the concepts of morality or social standards despite the generalized HIV prevalence [35]. This is supported by a study conducted in Malawi on adolescent girls and young women who reported low levels of vulnerability but suffered denial and delayed ART initiation after testing positive for HIV [33].

The establishment of the level of knowledge and perception of UTT is essential in facilitating designing and delivery of the context-specific educational program for the attainment of UNAIDS 95‐95‐95 targets. several participants from our study were not aware of UTT. This may further imply that such individuals were expecting continuous counselling after their HIV tests and not being initiated on ART on the same day of diagnosis. This outcome indicates the need for enhanced community awareness and health education on UTT and rapid ART initiation. This can be achieved through various modes of communication such as television advertisements, radio shows, and campaigns. Lessons drawn from a study conducted in the United States of America suggest that awareness of HIV testing can be successfully done using social media platforms and billboards [35].

The risk of attrition remains a challenge among men under “Treat All”; additional research is required on how ART delivery can be made efficient to link them in care. Most studies, including ours, have focused on patient-level factors on ART initiation. Grouping patients from different facilities together may cause loss of evidence on specific facility-level factors that influence ART initiation. This, therefore, calls for studies looking into the impact of health facility-level characteristics on ART initiation since performance might differ across facilities.

### Strengths and Limitations of this Study

- Our study was conducted in urban and peri‐urban communities, and this provides a reasonable basis for generalizability for the majority of people living with HIV in South Africa and sub-Saharan Africa. Participants were enrolled immediately after HIV diagnosis, allowing for observation of willingness to immediate ART initiation. However, our study was limited to adults and hence the results on characteristics of SDI and delayed ART initiators may not apply to infants and children. Our data collection was based on self-reported measures, which may have been subjective to social desirability and recall bias.

## Conclusion

The findings highlight several factors that influencing same-day ART uptake in eThekwini Municipality in line with national guidelines. The HIV status of the partner, knowledge of UTT and number of current sexual partners were identified as factors influencing uptake of SDI. Interventions to support client readiness for treatment uptake are therefore essential and emphasize a need to increase SDI ART initiation to avoid further transmission of the virus. The results also emphasize a vital need to not only streamline processes to increase immediate ART implementation/uptake further but also ensure individuals who test HIV positive receive adequate counselling in addressing the inhibiting factors.

## Supplementary Information

Below is the link to the electronic supplementary material.

Supplementary file1 (PDF 114 KB)

## Data Availability

The datasets used and/or analysed during the current study are available from the corresponding author on reasonable request.
