# Supplementary material for: Factors Influencing Rapid Antiretroviral Therapy Initiation at Four eThekwini Clinics, KwaZulu-Natal, South Africa
Source: AIDS Behav. 2021 Nov 15;26(6):1821–8. doi: 10.1007/s10461-021-03530-3 (PMC9046140; doi:10.1007/s10461-021-03530-3)
Supplement: Supplementary file 1 — Supplementary file1 (PDF 114 KB) [file 10461_2021_3530_MOESM1_ESM.pdf]

Acceptability and effectiveness of rapid ART initiation in the era of Universal Test and Treat: PATIENT BASELINE FORM

Today's Date:

yyyy-mm-dd hh:mm

Study ID:

Interviewer:

Facility:

Good morning/ afternoon my name is Sabina Govere. You are being invited to consider participating in a study titled "Acceptability and effectiveness of rapid ART initiation in the era of Universal Test and Treat by evaluating the impact on patient and healthcare workers." In this study we want to find out the factors that influence rapid and delayed ART uptake and how different PHC facilities in EThekwini Municipality are implementing the policy. We also want to assess the clinical outcomes of both early and delayed ART initiators. I will conduct the interview, record and take notes. I will ask you several questions. Your personal opinions and views are very important for us. There are no right or wrong answers. Please feel welcome to express yourself freely during the interview. This conversation will be recorded on tape. This is only for purpose of the research, only the lead researcher indicated on the consent form (and I) will listen to the tape. No names or personal information will be used in the report. There are no risks in participating in this study. We also seek your consent to check your clinic visits and results at six months from the day you initiated treatment. Participation is entirely voluntary: refusal to participate will not result in penalty and you can discontinue the study at any time, without penalty. You will not be denied any healthcare services that you are generally entitled to as a result of refusal or withdrawal from the study. Confidentiality: interviews will take place in a private place. All information given during the interview will be kept confidential. The interview will last for about twenty minutes. If you have any questions about the research, please contact the principal investigator, Sabina Govere, on 084 464 8815. If you have any questions or concerns regarding your rights as a research participant you may contact the Biomedical Ethics Research Office, UKZN, Private Bag X54001, Durban 4000. Telephone: 0027 (0) 31 260 4769/ 260 1074 Fax: 0027 (0) 31 260 4609 Administrator: Email: BREC@ukzn.ac.za

Participant: Name and Surname

I have been informed about the study entitled "Acceptability and effectiveness of rapid ART initiation in the era of Universal Test and Treat: evaluating the impact on patient and healthcare workers." I understand the purpose and procedures of the study. I have been given an opportunity to ask questions about the study and answered to my satisfaction.

PART I: DEMOGRAPHIC INFORMATION

Name and Surname

Date of Birth

yyyy-mm-dd

Age

Gender

- ◯ Male
- ◯ Female
- ◯ Transgender

South African ID

Marital Status

- ◯ Married
- ◯ Single
- ◯ Widowed
- ◯ Cohabitation
- ◯ Divorced
- ◯ Separated

Highest level of education

- ◯ None
- ◯ Primary School
- ◯ Some High School (but not Matric)
- ◯ Matric
- ◯ Certificate
- ◯ Diploma
- ◯ Degree

Employment Status

- ◯ Unemployed
- ◯ Self-employed
- ◯ Student
- ◯ Intern
- ◯ Employed

Do you have children of your own?

- ◯ Yes
- ◯ No

If yes, how many children?

____

PART II: HEALTH STATUS AND SEXUAL BEHAVIOUR

Have you ever been tested for HIV before today?

- ◯ Yes
- ◯ No

Have you ever tested POSITIVE for HIV before today?

- ◯ Yes
- ◯ No

If yes, when was the FIRST time you tested POSITIVE for HIV

yyyy-mm-dd

How many sexual partners did you have in the past 12 months?

Have you ever received treatment (ART) for HIV before today?

- ◯ Yes
- ◯ No

Has your sexual partner tested for HIV?

- ◯ No
- ◯ Unknown Status
- ◯ Yes, HIV negative
- ◯ Yes, HIV positive

**What was your perceived risk for getting HIV today?**	Definitely NOT going to acquire HIV	Probably NOT going to acquire HIV	Probably WILL become HIVinfected	Definitely WILL become HIVinfected
**What was your perceived risk for getting HIV today?**	◯	◯	◯	◯

PART III: KNOWLEDGE OF Universal Test and Treat AND ATTITUDES of ACCEPTABILITY

Did you know about Universal Test and Treat?

- ◯ Yes
- ◯ No
- ◯ Slightly

If yes, where did you hear about it?

- ◯ Clinic
- ◯ Family and friends
- ◯ Media

Date of HIV Test

yyyy-mm-dd

Date of ART initiation

yyyy-mm-dd

Have you accepted initiating on ART today?

- ◯ Yes
- ◯ No

Have you accepted being initiated on TLD?

- ☐ Yes
- ☐ No, i prefer TEE
- ☐ Any because I don't know much about the drugs

If yes, What do you think are the benefits?

- ☐ Starting before I get sick
- ☐ Live a normal longer life
- ☐ Suppressing my viral load
- ☐ To prevent transmitting to other uninfected people
- ☐ Pregnancy for PMTCT

If no, What are the reasons?

- ☐ Afraid of pills
- ☐ I'm still in shock and denial
- ☐ I feel Healthy
- ☐ Not ready today
- ☐ It wasn't explained clearly
- ☐ I will use traditional medicine
- ☐ I will go for prayers
- ☐ TB Presumptive

Will you disclose your status to someone?

- ◯ Yes
- ◯ No
- ◯ Noy yet sure

If yes, who will you disclose to?

- [ ] Parent
- [ ] Friend
- [ ] Family member
- [ ] Spouse/partner
- [ ] employer
- [ ] Church leader

Will you recommend rapid ART initiation to your family or friends?

- ( ) Yes
- ( ) No
- ( ) Maybe
